# Direct and Specific Effect of Sevoflurane Anesthesia on *rat Per2* Expression in the Suprachiasmatic Nucleus

**DOI:** 10.1371/journal.pone.0059454

**Published:** 2013-03-21

**Authors:** Megumi Anzai, Norio Iijima, Shimpei Higo, Ken Takumi, Izumi Matsuo, Keisuke Mori, Yumiko Ohe, Kana Kadota, Toshio Akimoto, Atsuhiro Sakamoto, Hitoshi Ozawa

**Affiliations:** 1 Department of Anatomy and Neurobiology, Graduate School of Medicine, Nippon Medical School, Bunkyo-ku, Tokyo, Japan; 2 Department of Anesthesiology, Graduate School of Medicine, Nippon Medical School, Bunkyo-ku, Tokyo, Japan; 3 Division of Laboratory Animal Science, Nippon Medical School, Bunkyo-ku, Tokyo, Japan; Vanderbilt University, United States of America

## Abstract

**Background:**

Our previous studies revealed that application of the inhalation anesthetic, sevoflurane, reversibly repressed the expression of *Per2* in the mouse suprachiasmatic nucleus (SCN). We aimed to examine whether sevoflurane directly affects the SCN.

**Methods:**

We performed *in vivo* and *in vitro* experiments to investigate rat *Per2* expression under sevoflurane-treatment. The *in vivo* effects of sevoflurane on *rPer2* expression were examined by quantitative *in situ* hybridization with a radioactively-labeled cRNA probe. Additionally, we examined the effect of sevoflurane anesthesia on rest/activity rhythms in the rat. In the *in vitro* experiments, we applied sevoflurane to SCN explant cultures from *Per2-dLuc* transgenic rats, and monitored luciferase bioluminescence, representing *Per2* promoter activity. Bioluminescence from two peripheral organs, the kidney cortex and the anterior pituitary gland, were also analyzed.

**Results:**

Application of sevoflurane in rats significantly suppressed *Per2* expression in the SCN compared with untreated animals. We observed no sevoflurane-induced phase-shift in the rest/activity rhythms. In the *in vitro* experiments, the intermittent application of sevoflurane repressed the increase of *Per2-dLuc* luminescence and led to a phase delay in the *Per2-dLuc* luminescence rhythm. Sevoflurane treatment did not suppress bioluminescence in the kidney cortex or the anterior pituitary gland.

**Conclusion:**

The suppression of *Per2-dLuc* luminescence by sevoflurane in *in vitro* SCN cultures isolated from peripheral inputs and other nuclei suggest a direct action of sevoflurane on the SCN itself. That sevoflurane has no such effect on peripheral organs suggests that this action might be mediated through a neuron-specific cellular mechanism or a regulation of the signal transduction between neurons.

## Introduction

General anesthesia, which results in unconsciousness and reduced pain perception, has been used clinically for over 150 years, and improvements in its safety have allowed many surgical advances [1]–[3]. Surprisingly, little is known about the mechanisms of general anesthesia, especially the molecular events induced by anesthetic agents in target cells and their effects on target proteins. Some studies have proposed that ligand-gated ion channels and G protein-coupled receptors are involved in general anesthesia [4], [5], but it is unclear whether these proteins alone are responsible for anesthetic effects. In a previous study, we comprehensively investigated the effects of general anesthesia on gene expression by microarray analysis and revealed that sevoflurane, currently the most widely used anesthetic for inhalation anesthesia, affected the expression of 1.5% of 10,000 genes in various rat organs [6]. *Period2* (*Per2),* a component of the “core loop” of the circadian clock [7], [8], was the only gene whose expression was reduced in the brain following anesthetic treatment. Although the brain is the major target organ of general anesthetics, the precise areas affected by these agents have not been determined. *Per2* is found in various areas of the brain, but is predominantly expressed in the suprachiasmatic nucleus (SCN) [9]. Therefore, we focused on *Per2* gene expression in the SCN under sevoflurane anesthesia. Our recent study using quantitative *in situ* hybridization revealed that mouse *Per2* (*mPer2)* expression in the SCN was repressed by sevoflurane-treatment [10], and the effect was most prominent when mice were anesthetized in the morning. Furthermore, only anesthetic treatment in the morning affected the subsequent *mPer2* expression cycle, which showed plasticity in the effect of anesthesia on gene expression [10], [11]. The aim of this study was to determine whether sevoflurane directly affect the SCN to suppress *Per2* expression or indirectly *via* other regions of the brain. We first investigated the effect of sevoflurane on individual rats, to examine whether the *in vivo* effect of sevoflurane on the rat *Per2* expression in the SCN was comparable to that on the mouse observed in our previous studies [10], [11]. Next we developed a system for applying inhalation anesthetics to cultured SCN slices isolated from extra-SCN neural inputs. Using this system, we examined whether the SCN was affected by *in vitro* application of sevoflurane. In previous studies of clock genes, transgenic mice (*mPer2 promoter::luciferase*) and rats (*mouse Per2 promoter-destabilized luciferase*; *Per2-dLuc*) have been used [12]–[15]. In this study, we chose the *Per2-dLuc* rat for our culture system. *Per2-dLuc* rat are widely used on the assumption that characteristics of the rat *Per2* promoter is similar to that of mouse *Per2*, based on the similarity in the sequence [16], [17]. Furthermore, we compared the *in vitro* effect of sevoflurane on the SCN with the effect on other peripheral organs that have circadian clocks [18], [19] to determine whether the effect of sevoflurane on Per2 expression has tissue specificity. If the target of the sevoflurane is a common part of the circadian machinery in the peripheral tissues and SCN, the sevoflurane sensitivity of peripheral tissues would be comparable to that of the SCN.

## Materials and Methods

### Animals

For *in situ* hybridization, male Wistar rats (8–10 weeks old) were purchased from Kiwa Laboratory Animals Japan, Inc (Tokyo, Japan). For analysis of locomotor activity and SCN explant cultures, we used *Per2-dLuc* transgenic male rats whose background strain was Wistar (8–10 weeks old) [15]. The founders of our *Per2-dLuc* rat colony were provided by Prof. Yasufumi Shigeyoshi (Kinki University School of Medicine, Sayama City, Osaka). Rats were housed in a 14 h light/10 h dark cycle (LD condition, with light from 06∶00 to 20∶00 h) at 21°C, with unrestricted access to food and water. All experiments in this study were carried out according to the National Institute of Health Guidelines for the Care and Use of Laboratory Animals. The Committee of Animal Research in Nippon Medical School also approved our experiments.

### Anesthetic Treatment of Individual Rats

Rats were placed in a chamber (40×27×17 cm) in complete darkness and exposed to a gas mixture of 4% sevoflurane (Maruishi Pharmaceutical, Osaka, Japan) and 40% O_2_ at a flow rate of 6 L/min. The temperature in the anesthesia chamber was maintained between 26–29°C during anesthetic treatment.

### Experiment 1: Measurement of Body Temperature and O_2_ Saturation

**Figure 1:**
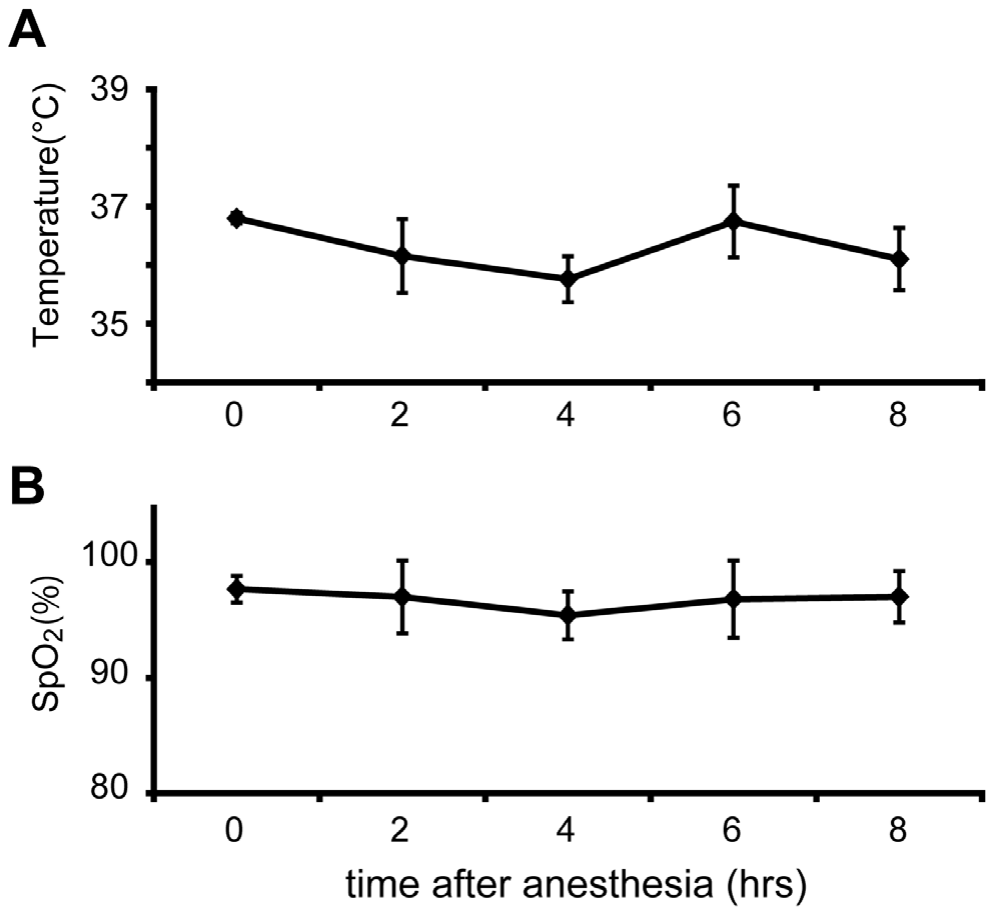
Measurements of body temperature and O_2_ saturation (SpO_2_). (A) The body temperature of rats was measured rectally before and during anesthesia. (B) The O_2_ saturation of rats was measured in the thigh during anesthesia. Values are expressed as means ± SD (n = 4).

Rats (n = 5) were exposed to sevoflurane from 08∶00–16∶00 (Fig. 1). Body temperature was measured rectally before, during and after anesthesia with a Digital Thermometer PTW-301 (Unique Medical, Tokyo, Japan). The O_2_ saturation was measured in the thigh during anesthesia with a Pulse Oximetry Vet Monitor Model 9847V (Nonin Medical Inc., Plymouth, MN, USA). These measurements were taken with the rats in the volatile sevoflurane flow within the chamber.

### Experiment 2: Analysis of Rest/activity Rhythm after Sevoflurane Anesthesia

**Figure 2:**
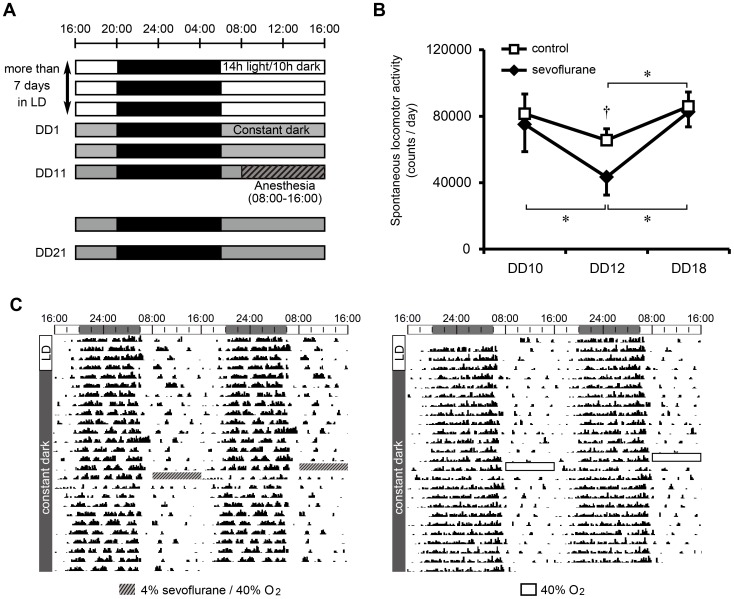
Effect of sevoflurane anesthesia on the rest/activity rhythm of rats. (A) The light/dark conditions, time of anesthetic treatment and time of sampling are indicated in the upper figure. White and black bars indicate light and dark periods, respectively. Gray bars correspond to the subjective day phase of the circadian clock. (B) Change in spontaneous locomotor activity induced by sevoflurane application. DD10, DD12, DD18 correspond to one day before application, one day after application, and 7 days after application, respectively. Data are mean ± SD. ^*^and ^†^denote statistically significant changes between days and between groups, respectively (repeated two-way ANOVA, *p*<0.05.). (C) A typical rest/activity rhythm in response to sevoflurane treatment (left) and 40% O_2_ without sevoflurane (right). Locomotor activity occurring every 10 min is double-plotted. Hatched gray rectangles and white boxes in the actograms indicate the time of sevoflurane treatment and 40% O_2_ application, respectively. The time of anesthetic treatment was 08∶00–16∶00.

Individual rats were placed in clear plastic cages (32×23×17 cm) and their locomotor activity was measured in 10 min bins using digital counters with passive infrared sensors (Supermex System; Muromachi Kikai, Tokyo, Japan). The activity monitoring cages were placed in a lightproof room. The light intensity on the room floor was 230 lux in the light condition. The experimental timeline is summarized in Fig. 2A. The rats were habituated to the experimental environment in the LD cycle. Thereafter, they were housed in constant darkness (DD condition: 14 h dark/10 h dark) throughout the experiment. Rats (n = 4) were anesthetized with sevoflurane for 8 h (08∶00–16∶00) on the 11th day after shifting to the DD condition (Fig. 2A) then returned to their original cages. Control rats were exposed to 40% O_2_ at flow rate of 6 L/min (n = 4). Transfer of rats between the cage and anesthesia chamber was performed under dim red light. Locomotor activity was monitored for more than 9 days after the anesthetic treatment. The data for each rat are summarized as an actogram (Fig. 2C), from which the anesthesia-induced phase-shift and the free-running period of the rest/activity rhythm pre- and post-anesthesia were extrapolated. The free-running period and phase-shifts were calculated under blind examination from visually fitted lines through activity onsets during the 10 days prior to, and 9 days following the treatment [11]. In the actogram, one day is defined as the hours from 16∶00 on a given day to 16∶00 on the following day. Locomotor activity was calculated for each day and compared against the day before (DD10), the day after (DD12) and 7 days after (DD18) anesthesia (Fig. 2B).

### Experiment 3: Analysis of Per2 mRNA Expression by in situ Hybridization

**Figure 3:**
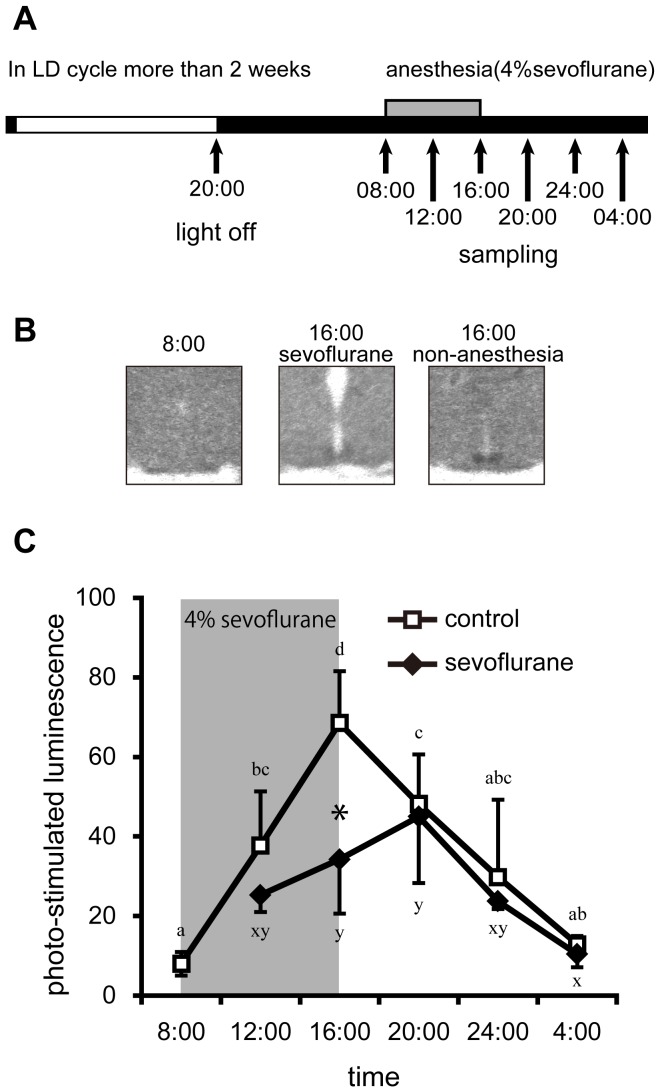
Effects of sevoflurane-anesthesia on *Per2* expression in the SCN *in vivo*. (A) The light/dark conditions, time of anesthesia and time of sampling in the experiment are illustrated. White and black bars indicate light and dark periods, respectively. Gray rectangles indicate the period of anesthetic treatment during the daytime (08∶00–16∶00). (B) Typical images of *in situ* hybridization. Labels indicate the sampling time and anesthetic treatment. (C) Intensity of *Per2* mRNA expression on the day of anesthetic treatment. Expression of *Per2* in non-treated (open squares) and treated (closed squares) rats in the DD condition. Data are mean ± SD. ^*^denotes a significant difference (two-way ANOVA, *p*<0.05) between the groups at the indicated time point. Same alphabetic annotation denotes non-significant difference between time points within the same group.

The experimental timeline is summarized in Fig. 3A. Rats were housed for at least two weeks to adapt to the standard LD cycle, then transferred to the DD condition prior to assay. For sampling, rats were exposed to sevoflurane from 08∶00–16∶00 in the dark. Samples were taken every 4 h, starting at 08∶00 and ending at 04∶00 (n = 4 at each time point for sevoflurane treatment, n = 4–8 for control).

For *in situ* hybridization, radiolabeled cRNA probes against *Per2* mRNA were made using [^35^S]-CTP (New England Nuclear, Boston, MA, USA) using a standard cDNA synthesis protocol. The cDNA fragments of *Per2* (1526 bp; corresponding to nucleotides 1390–2915 of NM_031678) were used as templates for *in vitro* transcription of cRNA probes. The *rPer2* fragment-containing vectors were kindly provided by Prof. Y. Shigeyoshi (Kinki University School of Medicine, Japan).

Rats were anesthetized with sodium pentobarbital (Kyoritsu Seiyaku Corporation, Tokyo, Japan, 70 mg/kg i.p.) under dim red light, then transcardially perfused with 50 mL of saline followed by a fixative containing 4% paraformaldehyde in 150 mL of 0.1 M phosphate buffer (PB) under light. The fixative perfusion was started within 2 min of turning on the light. Immediately after removal, brains were post-fixed in the same fixative for 24 h, and transferred into 0.1 M PB containing 20% sucrose for 48 h. Serial coronal sections (40-µm thick) were cut using a cryostat (Leica 3050, Heidelberg, Germany), collected into 4× standard saline citrate (SSC), and then processed for *in situ* hybridization as described previously [8]. Sections were treated with 1 µg/mL proteinase K (Sigma-Aldrich, St. Louis, MO, USA) in 10 mM Tris buffer (pH 7.4) and 10 mM EDTA for 10 min at room temperature. For quantitative analysis, the sections were hybridized with an [^35^S]-labeled cRNA probe. The hybridization buffer contained 10 mM Tris (pH 7.4), 60% formamide, 1× Denhardt’s solution, 10% dextran sulfate, 200 µg/mL tRNA, 600 mM sodium chloride, 1 mM EDTA and 0.25% sodium dodecyl sulfate (12h at 60°C). After hybridization, the sections were rinsed twice at 60°C in 2× SSC/50% formamide and treated with RNase (Roche Diagnostics, Mannheim, Germany). Then, they were mounted on gelatin-coated slides, dried, and dehydrated through a graded ethanol series. The slides were exposed to an imaging plate (IP: radiosensitive plates coated with BaFBr:Eu^2+^; Fuji Film BAS-SR) for 24 h.

*Per2* mRNA signals were quantified by measuring the photo-stimulated luminescence (PSL) in the SCN and the cerebral cortex using laser beam scanning, and data analyzed using FLA7000 (Fuji Film, Tokyo, Japan). After the PSL of the cerebral cortex in each section was determined (Fig. S1; no main effect of both time and sevoflurane treatment and no interaction between time and treatment was observed), this value was subtracted from the SCN PSL to obtain a background-corrected SCN PSL value. The intensities of the sections from the rostral to the caudal SCN (about 10 sections per rat brain) were added together, with the sum of these values representing the total amount of *Per2* mRNA in the SCN.

### Experiment 4: In vitro Study for Measurement of Bioluminescence from Slice Cultures

*Per2-dLuc* rats were sacrificed by decapitation under ether anesthesia between 09∶30 and 10∶30 (n = 5 each for the sevoflurane treatment and control). The brain, kidney and pituitary gland were removed and placed in ice-cold Hank’s balanced salt solution (HBSS: Thermo Fisher Scientific, Boston, USA). For the SCN culture, coronal brain sections (300 µm thick) were made with a microslicer DTK-3000 (D.S.K. Kyoto, Japan) and the sections containing the SCN were selected based on surrounding anatomical landmarks under a dissecting microscope. The SCN was then excised from the section using a scalpel blade. The kidney and anterior pituitary were prepared with the microslicer (200-µm thick sections). All tissues were then transferred onto Millicell cell culture inserts (PICMORG50, MILLIPORE, Billerica, MA, USA), which were placed in 35-mm tissue culture dishes containing 1.2 mL of DMEM media (Life Technologies, CA, USA ) supplemented with 1200 µg/mL sodium bicarbonate (Nacalai Tesque, Kyoto, Japan), 15 mM HEPES (DOJINDO, Kumamoto, Japan), 2% B-27 serum-free supplement, 20 µg/mL kanamycin (Life Technologies, CA, USA), 100 U/mL penicillin-streptomycin (Nacalai Tesque, Kyoto, Japan), 20 nM putrescine dihydrochloride, 5 µg/mL insulin, 100 µg/mL bovine apo-transferrin, 20 nM progesterone, 30 nM sodium selenite (Sigma-Aldrich, St Louis, MO, USA) and 0.5 mM luciferin potassium salt (Wako, Osaka, Japan). Slices were incubated at 34°C until assayed.

**Figure 4:**
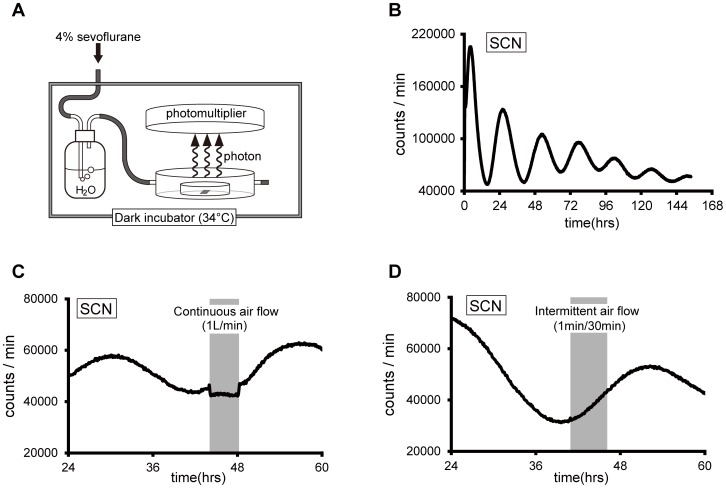
Development of the system for application of sevoflurane to the SCN culture slices. (A) The apparatus used to apply inhalation anesthetic. (B) Bioluminescence from the SCN slice without airflow. (C) Bioluminescence from the SCN with continuous airflow (1 L/min). (D) Bioluminescence from the SCN with intermittent application of airflow (1 L/min, for 1 min every 30 min).

For treatment of slice cultures with inhalation anesthetics, we made a glass chamber (radius: 2 cm, height: 1 cm) with two ducts for inflow and outflow (Fig. S2 and diagrammatically in Fig. 4A). Prepared tissue slices in 35-mm tissue culture dishes were placed in this chamber and exposed to an airflow with 4% sevoflurane at 1L/min *via* an SN-487-OT vaporizer (Shinano, Tokyo, Japan) after humidification and warming (Fig. 4A). For intermittent application of sevoflurane, we applied the anesthetic at 1L/min for 1 min every 30 min and sealed the anesthetic into the chamber. As a control, 1 L/min airflow without sevoflurane was applied. Bioluminescence was monitored in 6 min bins for 7 days using a Hamamatsu C9692-04 luminometer (Hamamatsu Photonix K.K., Shizuoka, Japan).

**Figure 5:**
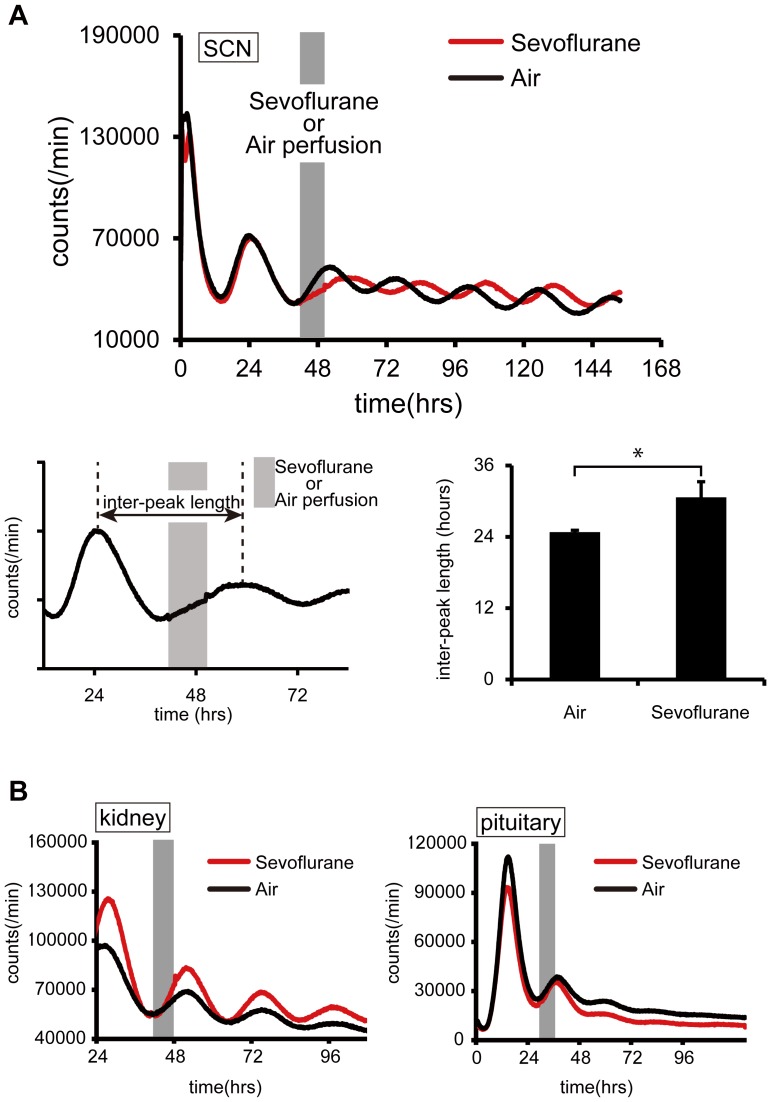
Responses of cultured tissue under anesthetic treatment. (A) The main panel shows the bioluminescence from an SCN slice during intermittent application of sevoflurane (red) or of airflow alone (black). The inset panel at the lower left demonstrates the definition of inter-peak length, while the inset lower right shows a comparison of inter-peak lengths under air and sevoflurane application. Data are mean ± SD (n = 5 for each group). ^*^denotes a statistically significant difference (Student’s t-test, *p*<0.05). (B) Bioluminescence from slices of kidney cortex (left) and the pituitary gland (right) with intermittent application of sevoflurane (red) or of airflow (black).

To examine whether sevoflurane application induced a phase-shift of the luminescence rhythm, we compared the inter-peak length of this rhythm in the sevoflurane-treated SCN with that in SCN exposed to airflow alone (Fig. 5A).

### Statistical Analysis

For body temperature and O2 saturation in Experiment 1, repeated one-way ANOVA was applied. For locomotor activity and day length of the rest/activity rhythm in Experiment 2 and Per2 quantification in Experiment 3, repeated two-way ANOVA with a post hoc Bonferroni test was applied. For phase-shift in Experiments 2 and inter-peak length in Experiment 4, Student’s t-test was applied. All statistical analyses were performed using IBM SPSS statistics software, with a *p*-value of less than 0.05 considered statistically significant.

## Results

### Stability of Body Temperature and O_2_ Saturation of Rats Under Sevoflurane Anesthesia (Experiment 1)

To exclude the possibility that changes in body temperature and/or O_2_ saturation might affect *Per2* expression, we monitored these parameters in rats under sevoflurane anesthesia. Rats receiving sevoflurane lost tail-clip responses within 30 min, and their body temperature was maintained between 35.36–38.42°C (mean ± SD = 36.30±0.45°C; Fig. 1A) throughout the 8 h anesthesia period. Similarly, O_2_ saturation remained stable between 95–100% (mean ± SD = 96.77±2.39%) during anesthesia (Fig. 1B). Rats regained responsiveness to handling a few minutes after cessation of anesthetic treatment. Neither body temperature nor O_2_ saturation showed any significant sevoflurane-dependent changes.

### Effects of Sevoflurane Anesthesia on the Circadian Rest/activity Rhythm of Rats (Experiment 2)

We observed no significant phase-shift in the rest/activity rhythm after sevoflurane treatment or control. No significant change in the free-running period of the rest/activity rhythm was observed for either condition (Fig. 2C and Fig. S3). In sevoflurane-treated rats, locomotor activity was significantly lower on the day after anesthesia (DD 12) than on the day before (DD 10), and 7 days after anesthesia (DD 18). On Day 12, the locomotor activity of sevoflurane-treated rats was significantly lower than that of control rats (Fig. 2B).

### Effects of Sevoflurane Anesthesia on the Circadian Expression of Per2 (Experiment 3)

Throughout a single sampling day, we observed a clear circadian expression of *Per2* in both sevoflurane-treated rats and controls. Two-way ANOVA revealed a significant main effects of both time and sevoflurane treatment individually, and a significant interaction between time and sevoflurane treatment. Immediately after anesthetic treatment (16∶00), *Per2* expression was significantly suppressed in treated rats compared with controls (Fig. 3B, C), but recovered to a level comparable to that in control rats by 20∶00 (after cessation of anesthesia).

### Development of the System for Application of Sevoflurane to the SCN Culture Slices (Experiment 4)

In the SCN explant culture experiments, the luciferase bioluminescence showed a clear rhythmic pattern with a period of about 24 h (Fig. 4B). Application of continuous airflow (1 L/min) at the point in the cycle where luminescence began to increase rapidly suppressed the increase in bioluminescence. The intensity of luminescence remained flat under constant airflow but recovered immediately after airflow was terminated (Fig. 4C). Under intermittent application of airflow (1 min, every 30 min), we observed no repression of bioluminescence (Fig. 4D). Although we observed robust circadian oscillation of bioluminescence in both the kidney and pituitary slices, the attenuation of this oscillation was faster in both these tissues than in the SCN slices. Unlike the SCN, the kidney slices displayed no repression or disturbance of bioluminescence under constant airflow (Fig. S4). To negate the effects of airflow on luminescence in cultured tissues, we employed intermittent application of sevoflurane in subsequent experiments.

### Effects of Sevoflurane Anesthesia on the Circadian Per2-dLuc Luminescence Rhythm in Cultured Tissues (Experiment 4)

Anesthetic treatment at the trough of the *Per2-dLuc* luminescence cycle reduced the increase in *Per2-dLuc* luminescence, resulting in a slower rate of increase to the peak value compared to slices in which only intermittent airflow with no sevoflurane was applied (Fig. 5A). The inter-peak length of the luminescent cycle with sevoflurane treatment (mean ± SD = 30.6±2.6 h) was longer than that with the application of airflow alone (24.8±0.3 h; Fig. 5A). The elongation of the inter-peak length caused by sevoflurane treatment resulted in a phase-shift of the luminescent cycle in sevoflurane-treated slices (Fig. 5A). No significant difference in the inter-peak length of subsequent cycles after treatment was observed between sevoflurane treatment (25.2±1.5 h) and air perfusion (24.9±1.3 h) (Fig. S5).

In peripheral tissues, the kidney cortex and the pituitary gland exhibited circadian rhythms that were less robust than those in the SCN. Bioluminescence in the kidney and pituitary gland was always diminished after 2–7 cycles (Fig. 5B), and when anesthetic treatment was initiated just prior to the increasing phase of the expression cycle, the bioluminescence of the kidney and the pituitary gland were not suppressed, the bioluminescence in these tissues was unchanged and we were unable to detect an obvious phase-shift (Fig. 5B).

## Discussion

Using the SCN slice culture system, we proved that sevoflurane had the suppressive effect on the expression of *Per2* in the rat SCN isolated from afferent inputs. This result indicates the direct action of sevoflurane on the SCN, and that the afferent inputs are not necessary for sevoflurane to affect the SCN. The SCN receives afferent projections from various neuronal populations including the retina, lateral geniculate body, the raphe nucleus [20]–[23] and processes the melatonin receptor, which tunes the circadian clock [24], [25]. Through inputs from these brain areas and the pineal gland, the circadian clock in the SCN is entrained with the environmental light/dark conditions. Although there are possibilities that sevoflurane can affect *Per2* expression in the SCN *via* these inputs *in vivo*, it is highly probable that the direct action we found in this study take a large role in sevoflurane effect on the SCN *in vivo*.

In order to investigate the effect of sevoflurane on isolated SCN, we developed the system for application of inhalation anesthetics to the cultured slices, and carefully validated the experimental condition using this system. Luciferase activity in the SCN was repressed by airflow alone, so we subsequently used an intermittent application of sevoflurane, and sealed the anesthetic into the chamber to minimize the effects of airflow. This system can be a useful tool for the experiment with inhalation anesthetics and gaseous reagents.

The rapid decrease in luminescence observed at the initiation of airflow (and the recovery at its termination) implies that airflow affects luminescence by modulating the availability of luciferin or ATP, rather than acting to regulate luciferase expression. The kidney cortex and anterior pituitary had no such airflow-sensitive mechanism.

Various anesthetics directly and competitively inhibit luciferase activity [26]–[28], an action thought to occur immediately after application of these agents. However, using intermittent application of anesthetics, we observed no obvious immediate repression of bioluminescence in the SCN, suggesting that competitive inhibition by anesthetics in our system is negligible. Bioluminescence was repressed in the SCN slice after 8 h of intermittent sevoflurane administration and, upon cessation of anesthesia, did not immediately recover to the intensity of the control. We also observed a phase-delay in the bioluminescence rhythm under these anesthesia conditions. These effects cannot be explained by competitive inhibition of luciferase by sevoflurane. We believe that the change in bioluminescence induced by intermittent application of sevoflurane reflects repression of *Per2* transcription activity, and indicates that sevoflurane directly affects the SCN to modify the phases of the circadian clock that involves the *Per2* promoter and PER2 protein.

Various peripheral tissues possess a circadian clock [18], [19], and we confirm here that the kidney cortex and anterior pituitary gland behave in a circadian fashion in our monitoring system. However, sevoflurane did not affect the phases of bioluminescence in these tissues, suggesting that these peripheral organs have no anesthetic-sensitive circadian machinery, yet our previous work demonstrated the effects of *in vivo* sevoflurane application on circadian clock-related genes in many peripheral organs [6]. Similarly, Kubo *et al* recently reported that *in vivo* application of 2,2,2-tribromoethanol induced a phase-shift in the rhythm of expression of *mPer2* in the liver [28]. The discrepancy between the result of research by Kubo et al and our current study may be depending on whether the investigations were performed *in vivo* or *in vitro*. It suggests that the circadian clock in peripheral organs could be affected indirectly, for example *via* the autonomic nervous system.

Assuming that the mechanism of the circadian clock is the same for the SCN and peripheral organs, we postulate that sevoflurane does not directly affect the common circadian clock machinery, comprising CLOCK, BMAL1, PERs and CRYs, as the peripheral organs do not respond to and are not modulated by anesthetics *in vitro*. A distinctive feature of the SCN in comparison with other peripheral organs is the close communication between its neurons, which leads to synchrony within the whole SCN [29]–[31]. Blockade of neuronal communication by tetrodotoxin drastically reduces *mPer2* as well as *mPer1* expression in the SCN [29], [32]. These reports indicate that intercellular communication associated with electrophysiological events is necessary for *Per1* and *Per2* expression. Most of the neurons in the SCN are GABAergic and express GABAA ionotropic receptors [30], [33]–[35]. GABAA receptor activation suppresses *mPer1* and *mPer2* expression in the SCN [36], [37]. Importantly, sevoflurane affects the GABAA receptor activation [38], [39]. It is therefore possible that sevoflurane represses *Per2* expression by regulating GABAA receptor in the SCN. Alternatively, sevoflurane could disrupt the cAMP signaling system, and thus repress cyclic AMP response element (CRE)-regulated gene expression [31]. Aton *et al* reported that inhibiting Gi/o with pertussis toxin abolished the rhythm of *mPer1* expression in most neurons [40], suggesting that if sevoflurane inhibits Gi signaling, *Per2* expression could be repressed in the SCN. Halothane, another inhalation anesthetic agent, disturbs SCN activity by blocking gap junctions [41], [42] and, although the effect of sevoflurane on gap junctions is not known, it is possible that sevoflurane could affect *Per2* expression in the SCN by a similar mechanism.

Our previous studies revealed that sevoflurane application to individual mice significantly and reversibly repressed *mPer2* expression in the SCN [10], [11]. We have replicated this finding in rats in the present study, which implies a common mechanism of sensitivity to sevoflurane. The peaks of luminescence intensity revealed a phase delay in the peak time of *Per2-dLuc* expression induced by anesthetic treatment in the rat SCN *in vitro* assay. However, in rest/activity rhythm, mice treated with sevoflurane showed only a 0.4 h phase-delay [10], [11] and a similar treatment in rats during the resting stage does not induce any phase-shift. Therefore, in both species, the modification of clock gene expression occurs in the near-absence of any phase-shift in the rest/activity rhythm, which is controlled by the circadian clock. This discrepancy is yet to be elucidated. One possibility is that the anesthesia-induced disturbance of the rhythm in *Per2* expression in the SCN was corrected by signals from other brain areas after cessation of anesthesia, resulting no phase-delay *in vivo*. We also observed a decrease in locomotor activity during the active phase after anesthesia in both mice [10] and rats, but are currently unable to determine whether this is caused by a disturbance in the circadian clock in the SCN or by an anesthetic effect in another area of the brain.

In this study, we focused solely on the *Per2* expression in the SCN, and the anesthetic effect on extra-SCN brain areas or other clock genes should be investigated for full understanding of the effect of anesthetics on the circadian clock. General anesthesia in humans occasionally causes atypical clinical signs, such as fatigue, sleep disorders, mood alteration and delirium, which have been linked with the clock genes [43]–[45]. We anticipate that our findings will help in understanding these symptoms in humans. Further research will be required to define the mechanism underpinning this chronopharmacology and to maximize the safety of anesthesia.

## Supporting Information

Figure S1 *Per2* expression in the cerebral cortex of sevoflurane-treated and control rats (closed and open squares, respectively). Data are mean ± SD.
(TIF)

Figure S2 Images show glass chamber used for application of anesthetics to cultured tissues in a cell culture insert (upper left), illustrating the ducts for perfusion and exhaust (lower left) and the chamber settled *in situ* in the luminometer (right).
(TIF)

Figure S3 Effect of sevoflurane on the free-running period and phase of rest/activity rhythms. (A) Free-running period of rest/activity rhythm in sevoflurane-treated rats (filled bar) and controls (open bar). Pre- and post-treatment values are the average of the DD1–DD10 and DD12–DD18, respectively. Data are mean ± SD (n = 4 for each group). (B) Quantitation of the phase-shift in sevoflurane-treated rats (filled bar) and controls (open bar).
(TIF)

Figure S4 Effect of continuous air flow on the bioluminescence of the kidney slices.
(TIF)

Figure S5 Inter-peak length in the subsequent cycle after sevoflurane treatment.
(TIF)
