# Supplementary figures and images for: Direct and Specific Effect of Sevoflurane Anesthesia on rat Per2 Expression in the Suprachiasmatic Nucleus
Source: PLoS One. 2013 Mar 21;8(3):e59454. doi: 10.1371/journal.pone.0059454 (PMC3605447; doi:10.1371/journal.pone.0059454)

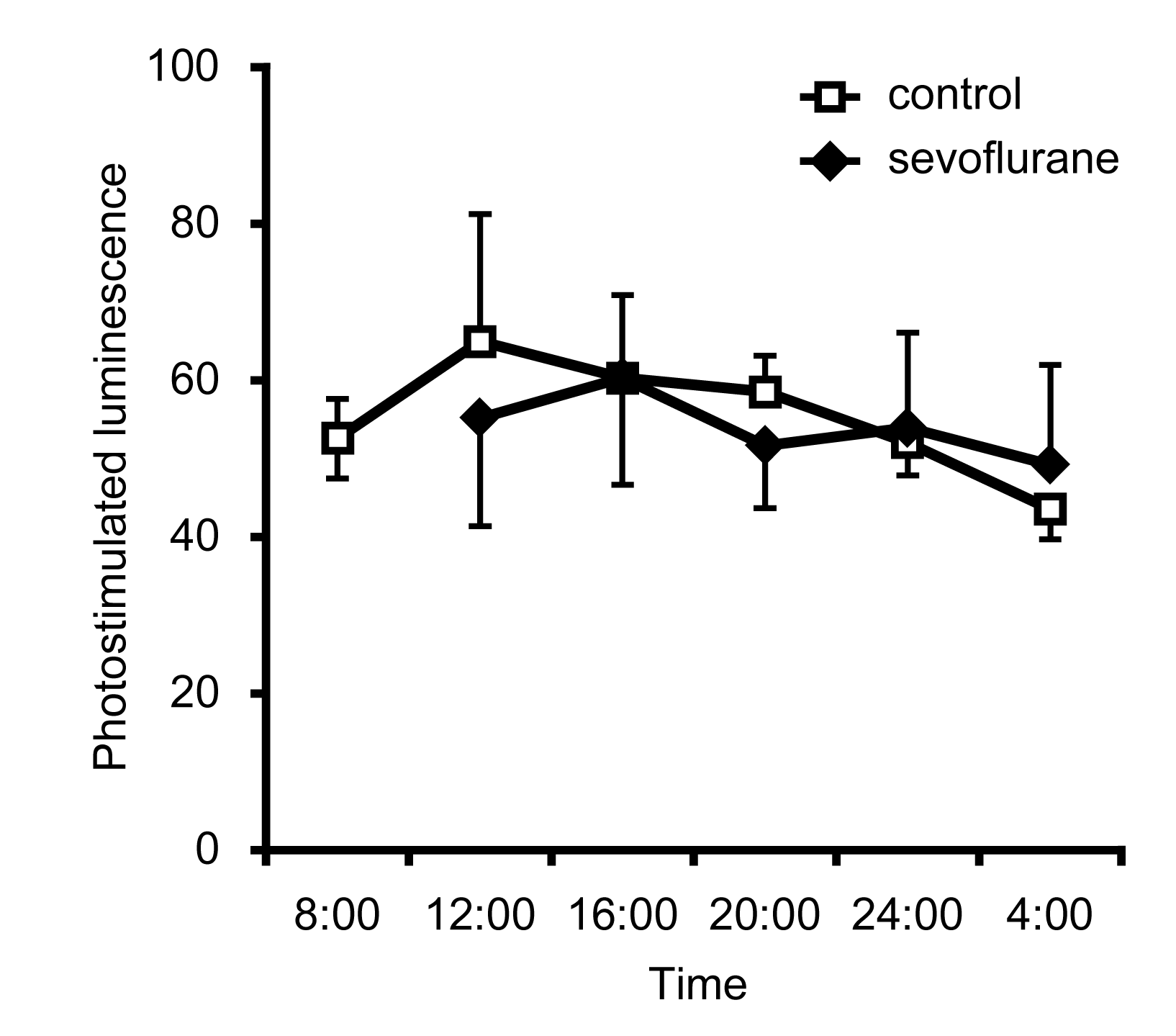

Supplement: Figure S1 — Per2 expression in the cerebral cortex of sevoflurane-treated and control rats (closed and open squares, respectively). Data are mean ± SD. (TIF) [file pone.0059454.s001.tif]

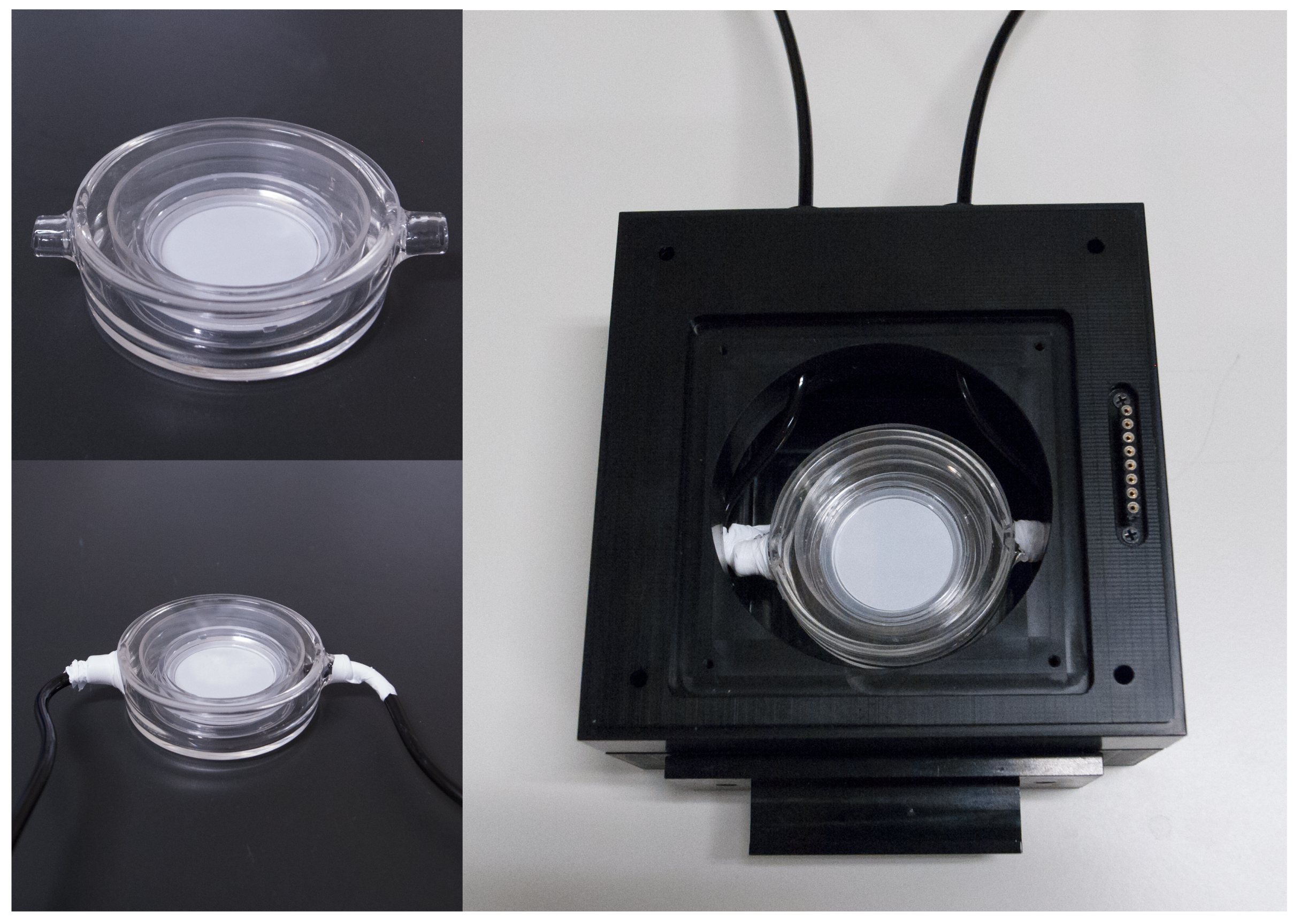

Supplement: Figure S2 — Images show glass chamber used for application of anesthetics to cultured tissues in a cell culture insert (upper left), illustrating the ducts for perfusion and exhaust (lower left) and the chamber settled in situ in the luminometer (right). (TIF) [file pone.0059454.s002.tif]

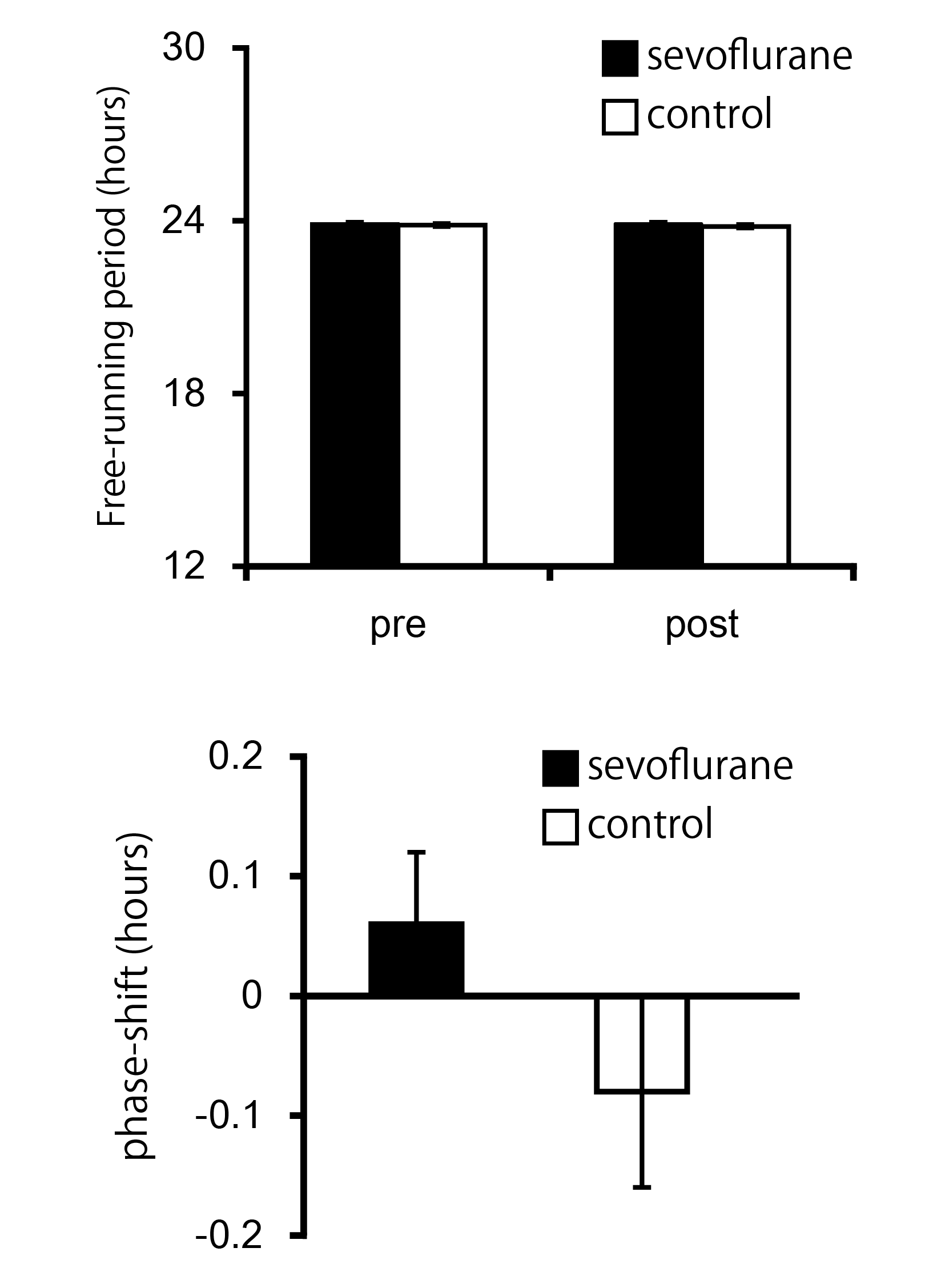

Supplement: Figure S3 — Effect of sevoflurane on the free-running period and phase of rest/activity rhythms. (A) Free-running period of rest/activity rhythm in sevoflurane-treated rats (filled bar) and controls (open bar). Pre- and post-treatment values are the average of the DD1–DD10 and DD12–DD18, respectively. Data are mean ± SD (n = 4 for each group). (B) Quantitation of the phase-shift in sevoflurane-treated rats (filled bar) and controls (open bar). (TIF) [file pone.0059454.s003.tif]

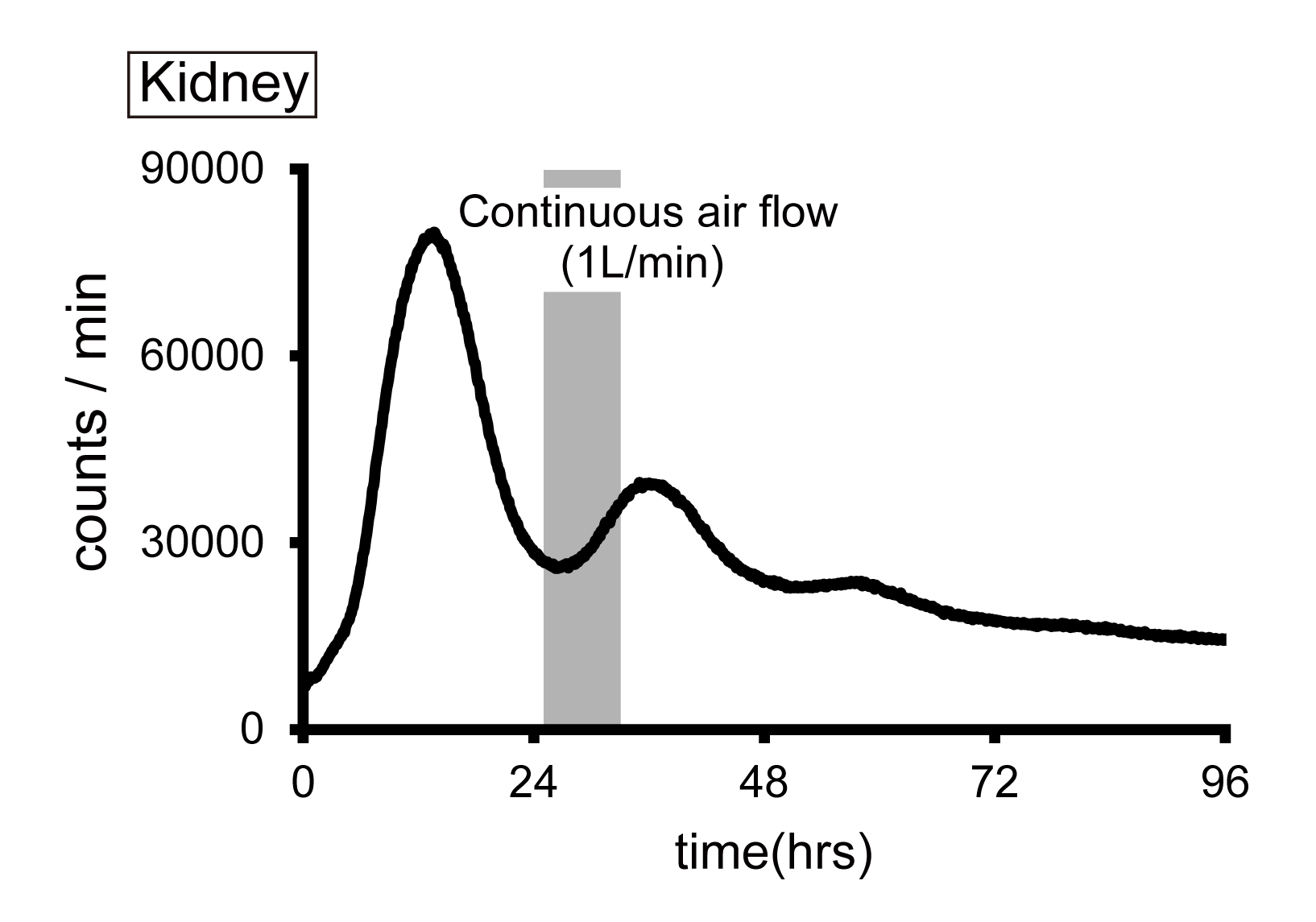

Supplement: Figure S4 — Effect of continuous air flow on the bioluminescence of the kidney slices. (TIF) [file pone.0059454.s004.tif]

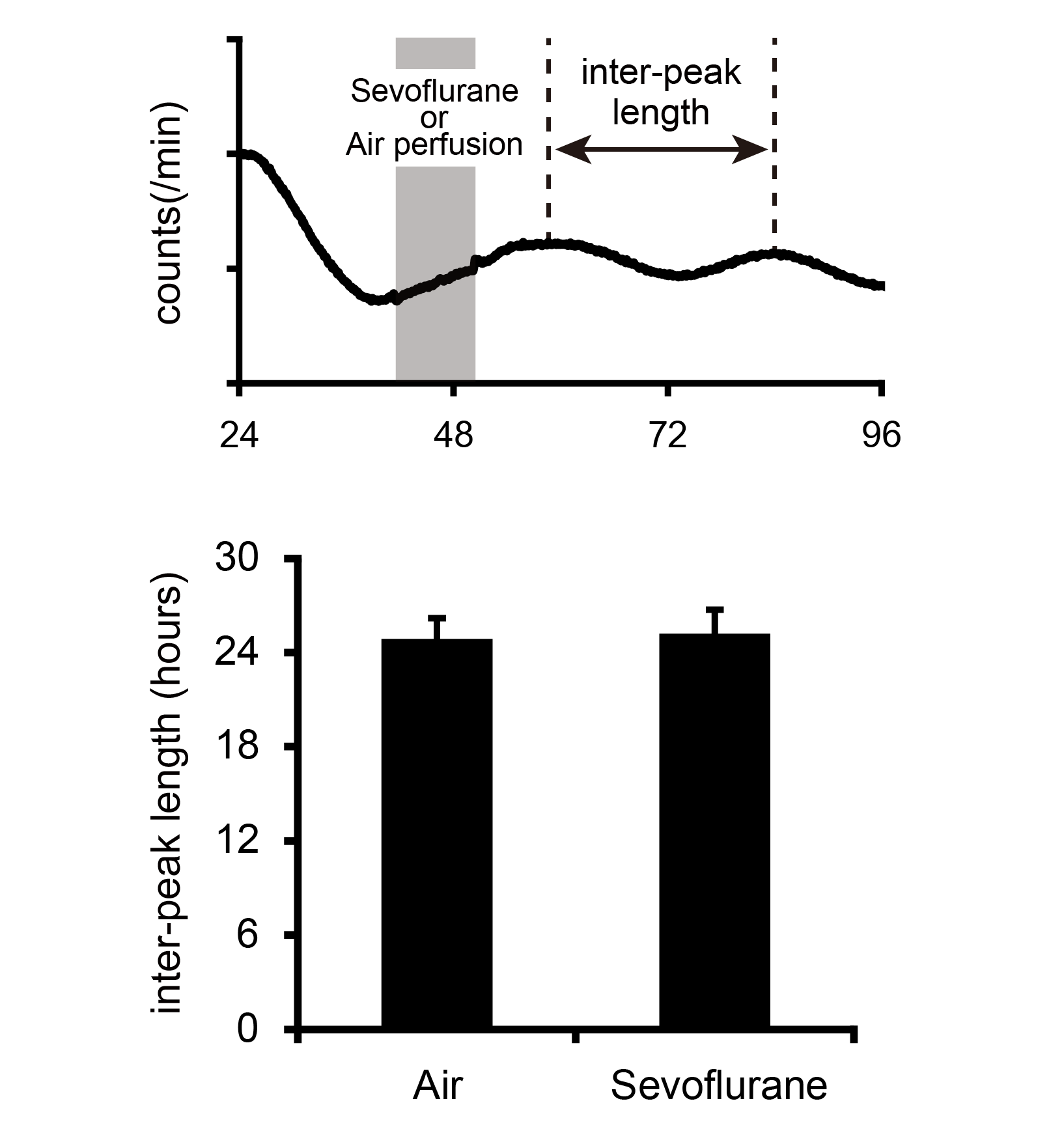

Supplement: Figure S5 — Inter-peak length in the subsequent cycle after sevoflurane treatment. (TIF) [file pone.0059454.s005.tif]
